# Functional brain alterations in Cushing’s syndrome

**DOI:** 10.3389/fendo.2023.1163482

**Published:** 2023-04-21

**Authors:** Eleni Papakokkinou, Oskar Ragnarsson

**Affiliations:** ^1^ Department of Internal Medicine and Clinical Nutrition, Institute of Medicine at Sahlgrenska Academy, University of Gothenburg, Gothenburg, Sweden; ^2^ Department of Endocrinology, Sahlgrenska University Hospital, Gothenburg, Sweden; ^3^ Wallenberg Center for Molecular and Translational Medicine, University of Gothenburg, Gothenburg, Sweden

**Keywords:** Cushing’s syndrome, functional connectivity, task-related functional magnetic resonance imaging, resting-state functional magnetic resonance imaging, prefrontal cortex, hippocampus, default mode network

## Abstract

Cognitive impairment and affective disorders are common in patients with Cushing’s syndrome (CS). In fact, as an effect of prolonged cortisol excess on the brain, patients with CS often have memory problems, concentration difficulties, impaired attention and executive function, that are not always reversible following successful treatment. Neuroimaging is essential for understanding the deleterious effects of hypercortisolism on the brain. In CS, structural alterations have been observed, including reduction of hippocampal volume, amygdala and the prefrontal cortex. The aim of this article is to summarize results from studies that have used functional magnetic resonance imaging (fMRI) to study functional brain alterations in patients with CS. In these studies, alterations in brain areas and networks essential for cognitive function, emotional processing, and executive function have been observed, both in patients with active CS as well as following treatment. Nevertheless, longitudinal studies with a comprehensive evaluation of functional brain alterations and neurocognitive evaluation are still needed to determine whether the apparent deleterious effects of hypercortisolism on the brain are reversible or not.

## Introduction

Cushing’s syndrome (CS) is caused by chronic and excessive exposure to cortisol (1). The most common causes of endogenous CS are Cushing’s disease (CD), i.e., adrenocorticotropic (ACTH)-producing pituitary adenoma, cortisol-producing adrenal adenoma and ectopic ACTH-producing tumors (2). Obesity, arterial hypertension, diabetes mellitus, myopathy, fractures, and depression are common and typical features of CS (3). Consequently, CS is associated with severely impaired quality of life, increased morbidity and mortality, not only during the stage of active hypercortisolism, but also during long-term follow-up after successful treatment (4–7).

Affective disorders and cognitive dysfunction are common manifestations of CS and have a great impact on quality of life in the patients (8). In fact, memory and concentration difficulties were among the earliest symptoms described in CS (9, 10). Recently, a more comprehensive cognitive dysfunction has been revealed in patients with active CS, including impaired attention, visuospatial processing, processing speed and executive functioning. Unfortunately, despite treatment, patients with CS still have impaired memory and executive function, in comparison to healthy controls, as well as when compared with patients treated for nonfunctioning pituitary macroadenomas (11). Also, patients with CS in long-term remission have impaired attention, working memory, verbal fluency, reading speed (12) and difficulties in decision making (13). Thus, it seems likely that the brain abnormalities are only partially reversible after successful treatment (14).

Glucocorticoid receptors are expressed globally throughout the whole brain (15). Hence, the whole brain is susceptible to excessive cortisol exposure in patients with CS. Mineralocorticoid receptors are also expressed in the brain, although distributed more specifically in the limbic system and the prefrontal cortex. However, since cortisol in high concentrations also binds to the mineralocorticoid receptors, the limbic system and prefrontal cortex, i.e., regions important for cognitive function, are especially vulnerable for hypercortisolism. Consequently, widespread alterations in white matter integrity have been observed in patients with CS (16) as well as structural changes in the hippocampus, amygdala and the prefrontal cortex (14, 17).

Functional magnetic resonance imaging (fMRI) has become an important method in investigating the detrimental effects of hypercortisolism on the brain. In this review, we focus on recent studies investigating brain alterations in patients with CS that have used fMRI during cognitive task performance or during a resting state (rsfMRI).

## Functional magnetic resonance imaging in Cushing’s syndrome

### Task-related fMRI

Task-related fMRI is used to examine functional alterations during performance of specific tasks, including cognitive tasks. Currently, four studies have evaluated brain activity in patients with CS with task-related fMRI (**Table 1** and **Fact box**). The first study was performed in twelve adolescent patients with active CS and showed increased activation in left amygdala and right anterior hippocampus during face encoding task, compared to healthy controls (28). These functional alterations were, however, not associated with affective disorders or memory impairment. Similar findings were found in adult patients with active CS, who demonstrated higher activation in the prefrontal cortex, hippocampus, thalamus and amygdala during identification of facial expressions (27). Of note, elevated activation in left middle frontal and lateral posterior/pulvinar areas, were associated with emotional processing, possibly indicating a compensatory activation (27). Likewise, these patients made more errors in identification of facial expressions and showed decreased activation in left anterior, superior temporal gyrus, a key area for facial emotional processing (27).

**Table 1:** Summary of studies in patients with Cushing’s syndrome using functional magnetic resonance imaging (fMRI).

Study	Design/ Origin	Subjects	Duration of remission (years)*	Method of fMRI	Main Findings	Comments
Li et al., 2022 (18)	Cross-sectional China	47 active CD 53 healthy controls	NA	rsfMRI	Altered hippocampal functional activity connectivity with default mode network, frontoparietal network, limbic networks in patients. The intrinsic hippocampal functional connectivity was associated with quality of life in patients.	Same cohort as Zhang et al., (19) and Wang et al., (20)
Zhang et al., 2021 (19)	Cross-sectional China	47 active CD 53 healthy controls	NA	rsfMRI and arterial spin labeling imaging Neurovascular coupling by using ratio of cerebral blood flow to functional connectivity strength	Changes of coupling cerebral blood flow to functional brain activity were observed in regions related to cognition in patients. Association was found between the regions with disrupted neurovascular coupling, and cognitive decline in patients.	Same cohort as Li et al., (18) and Wang et al., (20)
Xu et al., 2021 (21)	Cross-sectional China	38 active CD 33 CD in remission 41 healthy controls	0.2±0.04	rsfMRI graph theory approach	Lower connectivity in rich-club network in patients with active disease. After successful treatment with transsphenoidal surgery, the altered functional connectivity in the rich-club nodes was reversed.	
Wang et al., 2019 (20)	Cross-sectional China	32 active CD 32 healthy controls	NA	rsfMRI	Dysregulation of the normalized functional connectivity strengths (nFCSs) mainly in the default mode network in patients. Positive correlations between nFCS in the right parahippocampal cortex and morning serum cortisol levels.	Same cohort as Li et al., (18) and Zhang et al., (19)
Stomby et al., 2019 (22)	Cross-sectional Sweden, Norway	19 CS in remission 38 healthy controls	7 (6-10)	rsfMRI	Elevated resting state functional connectivity (RSFC) within the medial temporal lobe and prefrontal cortex networks in patients. Negative association between degree of elevated RSFC and duration of remission.	Same cohort as Ragnrasson et al., (23)
Ragnarsson et al., 2017 (23)	Cross- sectional Sweden, Norway	19 CS in remission 19 healthy controls	7 (6-10)	Task-related fMRI Episodic- and working- memory tasks	Lower functional brain responses especially in the prefrontal cortices in patients during episodic memory encoding and retrieval, as well as during working memory task.	Same cohort as Stomby et al., (22)
Jiang et al., 2017 (24)	Cross-sectional China	18 active CD 14 CD in remission 22 healthy controls	0.6±0.1	rsfMRI	Altered spontaneous brain activity in posterior cingulate cortex/precuneus, occipital lobe/cerebellum, thalamus, right postcentral gyrus and left prefrontal cortex) in patients with active CD. Correlation between altered spontaneous brain activity in prefrontal cortex and precuneus, and cortisol levels. Fewer brain regions were affected in patients in remission.	
van der Werff et al., 2015 (25)	Cross-sectional Netherlands	24 CD in remission 24 healthy controls	11±9	rsfMRI	Increased RSFC between the limbic network and anterior cingulate cortex in patients. Increased RSFC of the default mode network in patients. No differences in the executive control network.	Same cohort as Bas-Hoogendam et al., (26)
Bas-Hoogendam et al., 2015 (26)	Cross-sectional Netherlands	21 CD in remission 21 healthy controls	11±8	Task-related fMRI Emotional faces tasks	Lower activation of the medial prefrontal cortex and decreased coupling between medial prefrontal cortex and posterior cingulate cortex during processing of emotional faces in patients.	Same cohort as van der Werff et al., (25)
Langenecker et al., 2012 (27)	Cross-sectional USA	18 active CD 21 healthy controls	NA	Task-related fMRI Facial Emotion Perception Test	Patients made more errors in categorizing facial expressions and had less activation in left anterior, superior temporal gyrus.	
Maheu et al., 2008 (28)	Cross-sectional USA	12 adolescents with active CS 22 healthy controls	NA	Task-related fMRI Emotional faces encoding task	Increased activation in left amygdala and right anterior hippocampus in patients.	

CS, Cushing’s syndrome; CD, Cushing’s disease; fMRI, functional magnetic resonance imaging; NA, not applicable; nFCSs, normalized functional connectivity strengths; rsfMRI, resting-state fMRI; RSFC, resting state functional connectivity.

*Presented as mean ± standard deviation or median (interquartile range).

**Fact box**

**Table:** 

Default mode network	Includes several regions e.g., medial prefrontal cortex, posterior cingulate cortex and parietal regions.	Important for episodic memory, conceptual processing, self-referential processing.
Limbic network	Includes e.g., amygdala, hippocampus, parahippocampal gyrus, cingulate gyrus and associated structures.	Memory processing, emotional responses, fight-or flight responses.
Executive control network	Frontoparietal regions e.g., dorsolateral prefrontal cortex, anterior cingulate cortex, orbitofrontal cortex, lateral posterior parietal cortex.	Working memory, problem solving, task flexibility and decision-making.

**Figure 1:**
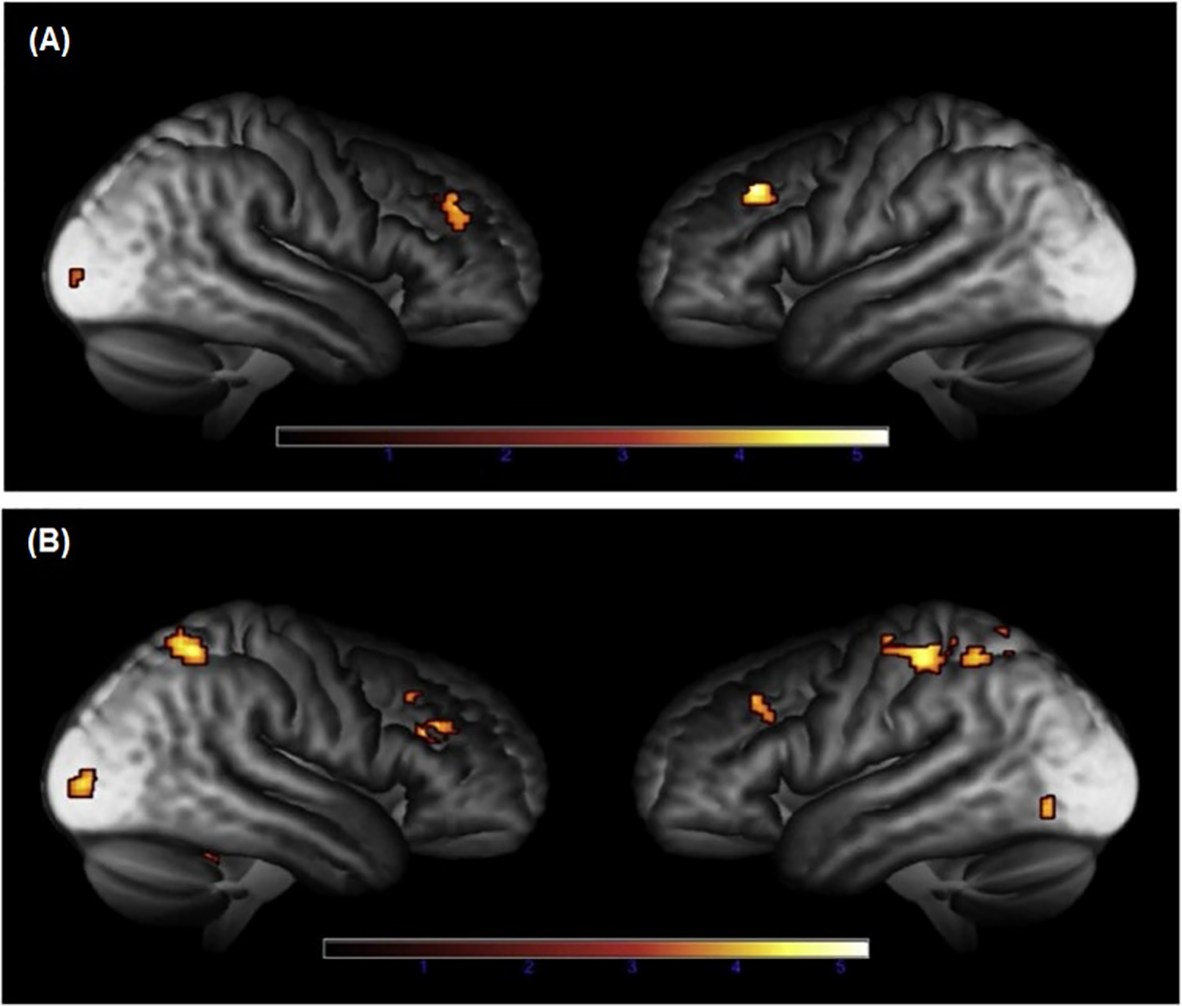
Brain areas with reduced functional brain responses during episodic memory during **(A)** encoding and **(B)** retrieval in patients with Cushing’s syndrome, compared to controls (23). Adapted from Ragnarsson O. et al. Psychoneuroendocrinology, 2017; 82:117-25, with permission from Elsevier.

Two additional studies showed that alterations in brain function are still present in patients with CS in long-term remission. In a study including twenty-one patients with CD in remission for a mean duration of eleven years, lower activation was shown in the medial prefrontal cortex during processing of facial expressions, as compared to controls (26). On the contrary, no alterations in amygdala activation were found during emotional processing. Interestingly, decreased functional coupling between the medial prefrontal cortex and the posterior cingulate cortex during emotional performance task was demonstrated (26), areas that are part of the default mode network which is important for episodic memory, conceptual processing, and self-referential processing (29). Finally, during working and episodic memory-task fMRI, decreased functional brain responses were found in prefrontal cortices in female patients with CS in long-term remission, compared to healthy controls matched for gender, age and education (23) (**Figure 1**).

### Resting-state fMRI

**Figure 2:**
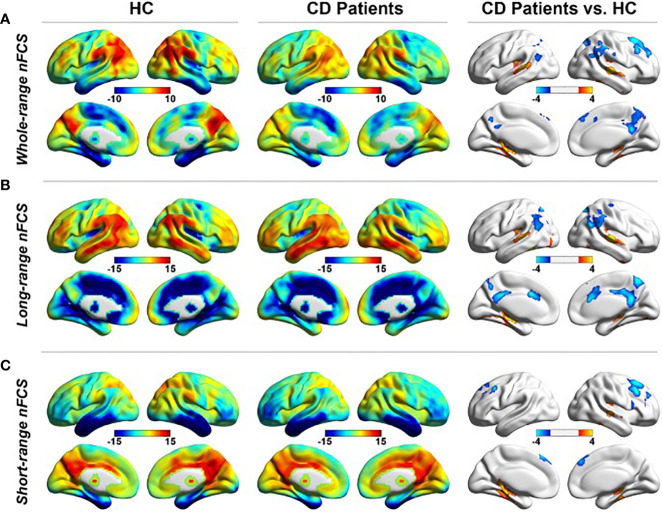
rsfMRI showing altered functional connectivity in the default mode network during **(A)** whole-range normalized functional connectivity strength (nFCS), **(B)** long-range nFCS, and **(C)** short-range nFCS, in patients with Cushing’s disease (CD) compared to healthy controls (HC) (20). Reproduced from Wang X. et al. Neuroradiology, 2019; 61 (8): 911-20, with permission from Springer Nature.

Seven studies have used rsMRI to study functional connectivity in CS (**Table 1**). During rsfMRI, the participants are asked to close their eyes and stay awake without performing any cognitive task. In active CD, widespread altered spontaneous brain activity was observed (24). More specifically, five brain regions were affected, including the posterior cingulate cortex/precuneus, occipital lobe/cerebellum, thalamus, right postcentral gyrus, and left prefrontal cortex. A significant correlation was shown between cortisol concentrations and altered spontaneous activity in prefrontal cortex/precuneus and the occipital lobe (24). Also, in patients with active CD, dysregulation of functional connectivity strength was found in the default mode network, including parahippocampal cortices, posterior cingulate cortices, lateral parietal cortices and right prefrontal cortex (20) (**Figure 2**). In the same cohort, altered hippocampal functional connectivity within the default mode network, frontoparietal and limbic network was found. The hippocampal functional activity correlated positively with quality of life (18). Moreover, disrupted connection between neurons and their vascular supply, measured as changes of coupling cerebral blood flow and functional connectivity strength, were observed in several brain regions related to cognitive function, suggesting an impact of hypercortisolism on the cerebral microenvironmental regulation (19). This disrupted neurovascular coupling was associated with cognitive impairment (19). Impaired functional connectivity was also found within the rich-club network, which consists of nodes with more dense interconnections, e.g., precuneus, cingulum and inferior temporal regions (21). After successful transsphenoidal surgery, the altered functional connectivity in the rich-club network was reversed (21).

Two studies have applied rsfMRI to explore brain functional networks in patients with CS in remission. The first study by van der Werff et al., included 24 patients with CD in long-term remission, and demonstrated increased functional connectivity between the limbic network and the anterior cingulate cortex, compared to matched healthy controls (25). In addition, increased resting-state functional connectivity was found in the default mode network in the left lateral occipital cortex (25). Nevertheless, these functional alterations were not associated with affective symptoms and no differences in the executive control network were observed, probably due to subtle cognitive impairment in the patients, and the absence of cognitive demands during sfMRI (25).

In line with this study, Stomby et al., showed elevated functional connectivity within the medial temporal lobe and prefrontal cortex in nineteen female patients with CS in long-term remission (22). On the contrary, reduced functional activity was found in the parietal lobe. Also, the degree of elevated functional connectivity in the medial temporal lobe was negatively associated with the duration in remission. Lack of neurocognitive evaluation of the participants did not allow further analysis of the association between altered functional activity and cognitive function.

## Relationship between functional brain alterations and neurocognitive assessment

Functional brain alterations in CS have mostly been observed in the default mode network and the limbic network. Whether these functional brain alterations are associated with cognitive deficits is still unclear. In studies where neurocognitive assessment has been performed, association between functional brain alterations and cognitive deficits or affective disorders, has not been distinctly confirmed (18, 23, 25, 26), probably due to small study populations and/or due to the use of insufficiently sensitive neurocognitive tests. Nevertheless, two studies have shown association between functional brain alterations and cognitive dysfunction. In the study by Langenecker et al., functional alterations in a region important for emotional processing were associated with worse performance in categorizing facial expressions (27). Also, Zhang et al. showed that functional alterations in areas of the executive control network were associated with cognitive decline (19). Moreover, it has previously been shown that patients with CS have impaired memory, concentration, attention as well as higher scores on the apathy scale (11, 12, 30). This is line with functional brain alterations observed in the default mode network and the limbic network.

All studies but one, were performed in adult patients and healthy controls matched at least for age and gender. In the only study including adolescents, functional brain alterations in amygdala and hippocampus were not associated with affective and memory impairments (28). Whether this reflects neural plasticity in younger patients, needs further investigation. Indeed, prospective longitudinal studies are needed to link functional brain alterations with neurocognitive deficits.

## Conclusion

Cognitive dysfunction is one of the most important issues that impacts quality of life negatively in patients with CS, even after successful treatment. fMRI is an essential tool that can be used to explore the potential mechanisms of cortisol excess on the brain. Functional brain alterations have been illustrated in the limbic network, default mode network and executive control network, namely networks that are essential for cognitive function. These findings strongly suggest an association between hypercortisolism, functional brain alterations and cognitive impairment in patients with CS. Nevertheless, the current studies do not provide robust information on whether the functional brain alterations reflect cognitive and affective symptoms in patients with CS. Moreover, altered functional connectivity and responses to performance tasks have been demonstrated in patients in remission, indicating persistent effects of cortisol excess on the brain despite successful treatment. Yet, the available data is not sufficient to elucidate the reversibility of the functional brain abnormalities in CS due to the small cohorts and lack of longitudinal follow-up. Hence, longitudinal prospective studies are needed to enable investigation of the course of functional brain alterations in patients with CS, from active hypercortisolism to long-term remission.

## Author contributions

All authors contributed to the article and approved the submitted version.
